# RETRACTION: Autism Spectrum Disorder Detection Using Facial Images: A Performance Comparison of Pretrained Convolutional Neural Networks

**DOI:** 10.1049/htl2.70095

**Published:** 2026-08-17

## Abstract

RETRACTION: I. Ahmad, J. Rashid, M. Faheem, A. Akram, N. A. Khan, and R. Amin, “Autism Spectrum Disorder Detection Using Facial Images: A Performance Comparison of Pretrained Convolutional Neural Networks,” *Healthcare Technology Letters* 11, no. 1 (2024): 227–239, https://doi.org/10.1049/htl2.12073.

The above article, published online on 08 January 2024 in Wiley Online Library (wileyonlinelibrary.com), has been retracted by agreement between the journal Editor‐in‐Chief, Christopher James; The Institution of Engineering and Technology; and John Wiley & Sons Ltd. The retraction was agreed upon due to concerns regarding both the reliability of the study and significant privacy issues. The dataset used in the study contains images of children purported to have, or not have autism spectrum disorder (ASD), sourced from the internet without any documented clinical history or independent verification of an ASD diagnosis, raising concerns about the scientific validity of the dataset. In addition, the images of minors have been used and presented in the article without consent from the individuals or their parents/legal guardians. For these reasons, the article has been retracted, and the corresponding images of children have been redacted. The authors have been informed of the decision, and they disagree with the retraction.

RETRACTION: I. Ahmad, J. Rashid, M. Faheem, A. Akram, N. A. Khan, and R. Amin, “Autism Spectrum Disorder Detection Using Facial Images: A Performance Comparison of Pretrained Convolutional Neural Networks,” *Healthcare Technology Letters* 11, no. 1 (2024): 227–239, https://doi.org/10.1049/htl2.12073.

The above article, published online on 08 January 2024 in Wiley Online Library (wileyonlinelibrary.com), has been retracted by agreement between the journal Editor‐in‐Chief, Christopher James; The Institution of Engineering and Technology; and John Wiley & Sons Ltd. The retraction was agreed upon due to concerns regarding both the reliability of the study and significant privacy issues. The dataset used in the study contains images of children purported to have, or not have autism spectrum disorder (ASD), sourced from the internet without any documented clinical history or independent verification of an ASD diagnosis, raising concerns about the scientific validity of the dataset. In addition, the images of minors have been used and presented in the article without consent from the individuals or their parents/legal guardians. For these reasons, the article has been retracted, and the corresponding images of children have been redacted. The authors have been informed of the decision, and they disagree with the retraction.

